# Comparison of Multiparametric Magnetic Resonance Imaging and Targeted Biopsy With Systematic Biopsy Alone for the Diagnosis of Prostate Cancer: A Systematic Review and Meta-analysis

**DOI:** 10.1001/jamanetworkopen.2019.8427

**Published:** 2019-08-07

**Authors:** Martha M. C. Elwenspoek, Athena L. Sheppard, Matthew D. F. McInnes, Samuel W. D. Merriel, Edward W. J. Rowe, Richard J. Bryant, Jenny L. Donovan, Penny Whiting

**Affiliations:** 1 National Institute for Health Research Collaboration for Leadership in Applied Health Research and Care West, University Hospitals Bristol National Health Service Foundation Trust, Bristol, United Kingdom; 2 Population Health Sciences, Bristol Medical School, University of Bristol, Bristol, United Kingdom; 3 Department of Radiology, The Ottawa Hospital, The University of Ottawa, Ottawa, Ontario, Canada; 4 Clinical Epidemiology Program, Ottawa Hospital Research Institute, Ottawa, Ontario, Canada; 5 Centre for Academic Primary Care, Population Health Sciences, Bristol Medical School, University of Bristol, Bristol, United Kingdom; 6 College of Medicine & Health, University of Exeter, Exeter, United Kingdom; 7 Bristol Urological Institute, Southmead Hospital, Bristol, United Kingdom; 8 Nuffield Department of Surgical Sciences, University of Oxford, Oxford, United Kingdom; 9 Department of Urology, Churchill Hospital, Oxford University Hospitals National Health Service Foundation Trust, Oxford, United Kingdom

## Abstract

**Importance:**

The current diagnostic pathway for patients with suspected prostate cancer (PCa) includes prostate biopsy. A large proportion of individuals who undergo biopsy have either no PCa or low-risk disease that does not require treatment. Unnecessary biopsies may potentially be avoided with prebiopsy imaging.

**Objective:**

To compare the performance of systematic transrectal ultrasonography–guided prostate biopsy vs prebiopsy biparametric or multiparametric magnetic resonance imaging (MRI) followed by targeted biopsy with or without systematic biopsy.

**Data Sources:**

MEDLINE, Embase, Cochrane, Web of Science, clinical trial registries, and reference lists of recent reviews were searched through December 2018 for randomized clinical trials using the terms “prostate cancer” and “MRI.”

**Study Selection:**

Randomized clinical trials comparing diagnostic pathways including prebiopsy MRI vs systematic transrectal ultrasonography–guided biopsy in biopsy-naive men with a clinical suspicion of PCa.

**Data Extraction and Synthesis:**

Data were pooled using random-effects meta-analysis. Risk of bias was assessed using the revised Cochrane tool. Preferred Reporting Items for Systematic Reviews and Meta-analyses (PRISMA) guidelines were followed. All review stages were conducted by 2 reviewers.

**Main Outcomes and Measures:**

Detection rate of clinically significant and insignificant PCa, number of biopsy procedures, number of biopsy cores taken, and complications.

**Results:**

Seven high-quality trials (2582 patients) were included. Compared with systematic transrectal ultrasonography–guided biopsy alone, MRI with or without targeted biopsy was associated with a 57% (95% CI, 2%-141%) improvement in the detection of clinically significant PCa, a 33% (95% CI, 23%-45%) potential reduction in the number of biopsy procedures, and a 77% (95% CI, 60%-93%) reduction in the number of cores taken per procedure. One trial showed reduced pain and bleeding adverse effects. Systematic sampling of the prostate in addition to the acquisition of targeted cores did not significantly improve the detection of clinically significant PCa compared with systematic biopsy alone.

**Conclusions and Relevance:**

In this meta-analysis, prebiopsy MRI combined with targeted biopsy vs systematic transrectal ultrasonography–guided biopsy alone was associated with improved detection of clinically significant PCa, despite substantial heterogeneity among trials. Prebiopsy MRI was associated with a reduced number of individual biopsy cores taken per procedure and with reduced adverse effects, and it potentially prevented unnecessary biopsies in some individuals. This evidence supports implementation of prebiopsy MRI into diagnostic pathways for suspected PCa.

## Key Points

**Question:**

Is prebiopsy magnetic resonance imaging combined with targeted biopsy associated with improved detection of clinically significant prostate cancer compared with transrectal ultrasonography–guided systematic prostate biopsy alone?

**Findings:**

This systematic review and meta-analysis of 7 randomized clinical trials (2582 patients) demonstrates that prebiopsy magnetic resonance imaging combined with targeted biopsy is associated with improved detection of clinically significant prostate cancer and reduced numbers of biopsy cores per procedure, while potentially avoiding unnecessary biopsies.

**Meaning:**

These findings support the introduction of prebiopsy magnetic resonance imaging into the diagnostic pathway for biopsy-naive men with suspected prostate cancer.

## Teaser

This systematic review and meta-analysis investigates whether magnetic resonance imaging combined with targeted biopsy is associated with improved detection of clinically significant prostate cancer compared with transrectal ultrasonography–guided systematic prostate biopsy alone.

## Introduction

Prostate cancer (PCa) is the most commonly diagnosed cancer in men and the second leading cause of cancer-associated death among men in the United States.^1^ Despite this statistic, a large number of PCas are not clinically significant and are unlikely to lead to problems if left untreated.^2^ Distinguishing high-risk from low-risk PCa remains difficult,^3^ leading to overdiagnosis and, for some men, unnecessary invasive treatments and treatment-associated morbidity.^4^ There is, therefore, an unmet clinical need to develop tests that can detect clinically significant PCa (csPCa) while reducing overdiagnosis of low-risk disease.

Clinical findings of possible PCa include elevated prostate-specific antigen (PSA) levels and/or abnormal digital rectal examination findings. The US Preventive Services Task Force,^5^ European Association of Urology,^6^ and UK National Institute for Health and Care Excellence^7^ recommend transrectal ultrasonography (TRUS)–guided biopsy as a standard investigation in the diagnosis of PCa. Transrectal ultrasonography is primarily used for anatomical guidance during biopsy, with approximately 10 to 14 individual biopsy cores taken systematically from the prostate (depending on the gland volume). However, a TRUS-guided systematic biopsy predominantly samples the peripheral zone of the prostate gland, so some PCa foci may be missed or undersampled, leading to disease misclassification and/or underdiagnosis.^8^

A recent development in the diagnostic pathway for suspected PCa involves prebiopsy magnetic resonance imaging (MRI) using 2 or more parameters to identify suspicious areas. Multiparametric MRI (mpMRI) uses T2-weighted, dynamic contrast-enhanced, and diffusion-weighted imaging, whereas biparametric MRI only uses T2-weighted and diffusion-weighted imaging. These MRI-visualized lesions are graded using the Prostate Imaging Reporting and Data System^9^ and can be specifically targeted at biopsy. This method offers potential advantages over a pathway where only peripheral zone cores are taken systematically without prior imaging, including more-accurate detection of csPCa using targeted biopsy, the possibility of reducing the need for a biopsy in some individuals with normal MRI findings, and a potential reduction in the number of biopsy cores taken per procedure. Avoiding unnecessary biopsies may reduce serious adverse events associated with this procedure, such as bleeding, sepsis, and, rarely, death.^10^ Fewer biopsy cores being taken per procedure could reduce the total procedure time and may reduce the risk of adverse effects, making it a more acceptable investigation for patients.^11^ Previous studies^12^ have suggested that using prebiopsy mpMRI to guide biopsies may increase the sensitivity to detect higher-grade PCa appropriate for treatment. Prebiopsy mpMRI has recently been recommended in the United Kingdom as the standard of care for biopsy-naive patients with suspected PCa.^13^

**Figure 1.**
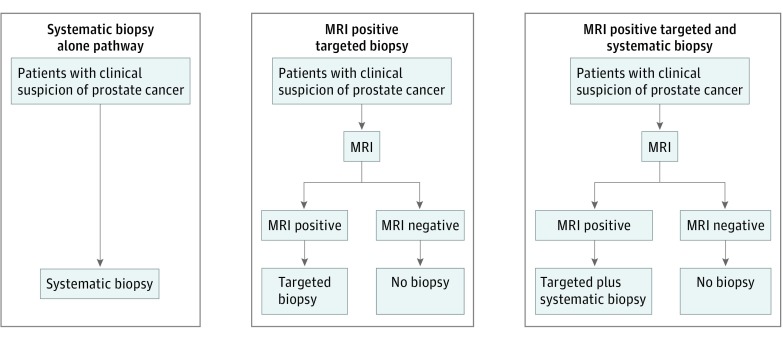
Three Diagnostic Pathways Used to Detect Clinically Significant Prostate Cancer Flowcharts show, from left to right, a transrectal ultrasonography–guided systematic biopsy alone pathway (control), in which all patients with clinical suspicion of prostate cancer undergo this procedure; a magnetic resonance imaging (MRI) plus targeted biopsy pathway, in which individuals with a positive prebiopsy MRI undergo a transrectal ultrasonography–guided targeted biopsy alone; and an MRI plus targeted and systematic biopsy pathway, in which individuals with positive prebiopsy MRI findings undergo a transrectal ultrasonography–guided targeted biopsy combined with a systematic biopsy. In both hypothetical MRI pathways, individuals with negative MRI findings do not undergo a prostate biopsy procedure.

Evidence supporting the value of introducing MRI into the diagnostic pathway for suspected PCa is increasing. Several randomized clinical trials (RCTs) have been conducted comparing a systematic TRUS-guided biopsy pathway (ie, systematic biopsy alone) with pathways including a prebiopsy MRI. We conducted a systematic review of these RCTs and investigated 2 different prebiopsy MRI pathways: (1) prebiopsy MRI followed by a targeted biopsy only (ie, MRI plus targeted biopsy pathway) and (2) prebiopsy MRI followed by a biopsy obtaining both targeted and systematic biopsy cores (ie, MRI plus targeted and systematic biopsy pathway) (Figure 1). Our main outcome was the detection rate of csPCa. Secondary outcomes were the detection rate of any-grade PCa, the number of biopsy procedures potentially avoided, the number of any-grade PCa missed by MRI, and complications.

## Methods

This review followed recommended methods for systematic reviews^14,15^ and is reported according to the Preferred Reporting Items for Systematic Reviews and Meta-analyses (PRISMA) reporting guideline. We expanded the data extraction and analysis, as described elsewhere,^16^ to differentiate between the 2 prebiopsy MRI pathways and to include the secondary outcome of PCa missed by MRI.

### Data Sources and Study Selection

Randomized clinical trials including biopsy-naive men with clinical suspicion for PCa that compared a 2-step MRI pathway (prebiopsy MRI group) with TRUS-guided systematic biopsy (systematic biopsy alone group) were eligible for inclusion. Eligible MRI pathways consisted of prebiopsy MRI using 2 or more parameters, followed by a targeted biopsy with or without systematic sampling based on the MRI results (MRI plus targeted biopsy or MRI plus targeted and systematic biopsy). MEDLINE, Embase, Cochrane, and Web of Science were searched through December 2018 using the terms “prostate cancer” and “MRI” and an RCT filter.^16^ Trial registries and reference lists of recent reviews were also searched. Abstracts and full texts were independently screened by 2 reviewers using Rayyan.^17^ Any discrepancies between the reviewers were resolved through discussion or referral to a third reviewer.

### Data Extraction and Risk of Bias Assessment

Data were extracted by 1 author and checked by a second author using standardized data extraction forms. Data on patient characteristics, study design, imaging, and biopsy protocols were extracted according to the Standards of Reporting for MRI-Targeted Biopsy Studies recommendations.^3^ We investigated 2 hypothetical prebiopsy MRI pathways and extracted data that allowed analysis of these pathways (Figure 1): (1) where prebiopsy MRI–positive patients undergo targeted biopsy alone (MRI plus targeted biopsy pathway), or (2) where prebiopsy MRI–positive patients undergo biopsy including targeted and systematic cores (MRI plus targeted and systematic biopsy pathway). In RCTs that investigated the MRI plus targeted and systematic biopsy pathway, but also reported data that allowed deduction of outcomes for MRI plus targeted biopsy (ie, trials that reported results for the targeted and systematic cores separately), data were extracted for both potential prebiopsy MRI pathways. We extracted the number of patients with a diagnosis of csPCa or clinically insignificant PCa according to the definition of clinical significance used in each RCT (eTable 1 in the Supplement). The number of patients with negative MRI findings was extracted to determine the number of biopsy procedures that could potentially have been avoided. We also extracted information on those cancers missed according to the systematic TRUS-guided biopsy or a reference standard, such as prostatectomy or saturation biopsy. These numbers were used to calculate percentages of cancers missed by MRI (ie, when the MRI findings were negative, but a cancer was subsequently identified at systematic biopsy, prostatectomy, or saturation biopsy) or by targeted biopsy alone (ie, when the targeted cores did not sample the cancer, but when the malignant neoplasm was identified within systematic cores). Risk of bias was assessed using the revised Cochrane tool (RoB 2.0 tool).^18^ Authors were contacted to provide missing information.

### Data Synthesis and Analysis

Random-effects meta-analysis models were used to estimate summary effect estimates (risk ratios and percentages) and to allow for variation among studies using the method of DerSimonian and Laird.^19^ Heterogeneity was assessed using the *I*^2^ statistic.^20^ Ninety-five percent confidence intervals around risk ratios were calculated using the Woolf method, and 95% confidence intervals around percentages were calculated using the exact binomial (Clopper-Pearson) procedure.^21^ A *P* < .05 was regarded as statistically significant (1-sided χ^2^ test). All analyses were performed in Stata statistical software version 15.1 (StataCorp)^22^ using the metan and metaprop commands.^23,24^

Summary risk ratios were estimated to compare the proportion of csPCas detected for each prebiopsy MRI pathway (MRI plus targeted and systematic biopsy and MRI plus targeted biopsy) compared with the systematic biopsy alone group. We stratified the analysis by biparametric MRI and mpMRI given the fundamental differences in these MRI techniques. We also estimated the summary percentage of patients with negative MRI findings (ie, potential biopsies avoided) with any-grade PCa and csPCa cases missed by prebiopsy MRI or targeted biopsy alone.

## Results

**Figure 2.**
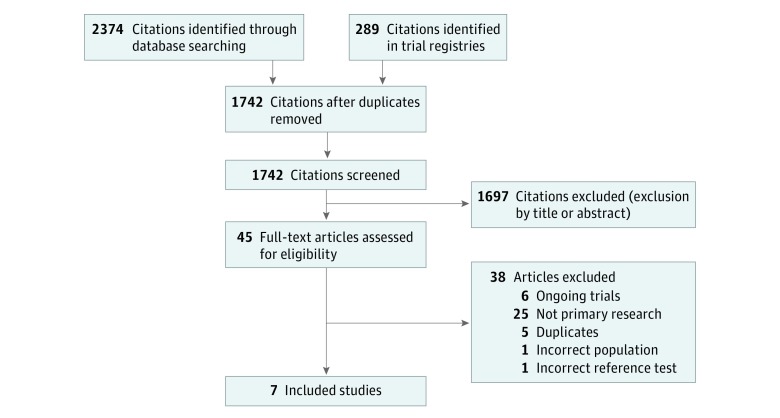
Diagram of Inclusion Criteria for Randomized Clinical Trials

The literature searches identified 1742 records, of which 7 RCTs fulfilled the inclusion criteria (Figure 2): 6 original investigations^25,26,27,28,29,30^ and 1 conference abstract^31^ including 2582 patients in total. In 5 RCTs,^25,26,27,28,31^ the clinical suspicion of PCa was based on elevated PSA levels, abnormal digital rectal examination findings, or both. In 2 RCTs,^29,30^ patients with abnormal digital rectal examination findings were excluded. Two RCTs^25,29^ applied an age restriction excluding patients older than 75 years. There were no significant differences in age, prostate volume, or prebiopsy PSA levels between individuals in the prebiopsy MRI pathways and those in the systematic biopsy alone group, although 1 trial^27^ did not report these measures (Table).

**Table.** Study Characteristics

Source	Dates of Recruitment	Inclusion Criteria	Exclusion Criteria	Men Randomized, No.			Age, y			Prostate Volume, mL			Prebiopsy PSA Level, ng/mL		
				Total	MRI^a^	Standard^b^	Overall	MRI^a^	Standard^b^	Overall	MRI^a^	Standard^b^	Overall	MRI^a^	Standard^b^
Baco et al,^25^ 2016 Norway	Sep 2011-Jun 2013	Age <75 y; clinical suspicion of PCa, based on verified PSA level increase to 4-20 ng/mL, abnormal DRE findings, or both	Previous prostate biopsy or MRI of the prostate; contraindication to MRI	183	90	93	65 (59-69)^c^	64 (58-69)^c^	65 (59-69)^c^	42 (30-59)^c^	45 (33-60)^c^	40 (29-52)^c^	7.3 (5.5-9.9)^c^	6.9 (5.2-9.2)^c^	7.6 (5.9-10.4)^c^
Kasivisvanathan et al,^26^ 2018 United Kingdom	Feb 2016-Aug 2017	Clinical suspicion of PCa, based on elevated PSA level, abnormal DRE findings, or both; PSA level ≤20 ng/mL	Previous prostate biopsy or treatment for prostate cancer; DRE findings that suggest extracapsular disease; contraindications to biopsy or MRI	500	252	248	64.4 (7.8)^d^	64.4 (7.5)^d^	64.5 (8.0)^d^	Not reported				6.75 (5.16-9.35)^c^	6.50 (5.14-8.65)^c^
Panebianco et al,^27^ 2015 Italy	Oct 2011-Mar 2014	Symptoms highly suggestive of PCa; total PSA level >4 ng/mL; PSA density >0.15; PSA velocity >0.75 ng/mL/y; free/total PSA ratio <0.10 when total PSA level was 4-10 ng/mL	Previous prostate biopsy	1140	570	570	64 (51-82)^e^			Not reported			Not reported		
Park et al,^28^ 2011 Korea	Jul 2008-Dec 2009	Clinical suspicion of PCa, based on high PSA level or abnormal DRE findings	Previous prostate biopsy or treatments for prostate cancer	103	54	49	62 (37-92)^e^	63 (40-82)^e^	61 (37-92)^e^	37 (15-94)^e^	37 (17-94)^e^	38 (15-87)^e^	5.8 (2.9-9.9)^e^	6.1 (4.0-9.7)^e^	5.6 (2.9-9.9)^e^
Plata-Bello et al,^31^ 2018 Spain	Feb 2015-Oct 2017	Clinical suspicion of PCa, based on elevated PSA level (4-20 ng/mL), abnormal DRE findings, or both	Previous prostate biopsy	303	182	121		67.9 (8.5)^d^	67.6 (8.8)^d^		47.5 (26.0)^d^	53.5 (25.5)^d^		6.48 (2.60)^d^	7.74 (6.87)^d^
Porpiglia et al,^29^ 2017 Italy	Nov 2014-Mar 2016	Aged ≤75 y; clinical suspicion of PCa; PSA level ≤15 ng/m findings; negative DRE findings	Previous prostate biopsy or surgery; previous prostate MRI; contraindication to MRI	223	111	112		64 (58-70)^c^	66 (60-70)^c^		46.2 (34.5-71.6)^c^	45.7 (34.6-65.0)^c^		5.9 (4.8-7.5)^c^	6.7 (5.5-8.5)^c^
Tonttila et al,^30^ 2016 Finland	Apr 2011-Dec 2014	Clinical suspicion of PCa, based on elevated PSA level (PSA<20 ng/mL or free-to-total PSA ratio ≤0.15 and PSA<10 ng/mL in repeated measurements); no evidence of PSA level increase due to noncancerous factors (ie, urinary tract infection); negative DRE findings	Previous prostate biopsy or surgery; contraindication to MRI	130	65	65		63 (60-66)^c^	62 (56-67)^c^		27.8 (23.5-36.6)^c^	31.8 (26.1-44.3)^c^		6.1 (4.2-9.9)^c^	6.2 (4.0-10.7)^c^

Abbreviations: DRE, digital rectal examination; MRI, magnetic resonance imaging; PCa, prostate cancer; PSA, prostate-specific antigen.

SI conversion factor: to convert PSA to μg/L, multiply by 1.0.

^a^ MRI pathway (intervention group).

^b^ Standard pathway (comparator group).

^c^ Values are median (interquartile range).

^d^ Values are mean (SD).

^e^ Values are mean (range).

**Figure 3.**
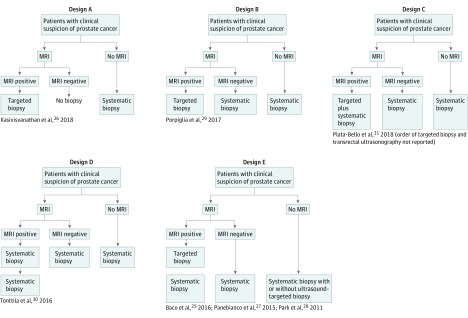
Study Designs of the Included Randomized Clinical Trials Designs A and B allowed for sufficient data extraction to analyze the systematic biopsy alone pathway vs the magnetic resonance imaging (MRI) plus targeted biopsy pathway. Design C allowed for sufficient data extraction of the systematic biopsy alone and the MRI plus targeted and systematic biopsy pathways, but not the MRI plus targeted biopsy pathway because separate data were not reported for the content of targeted and systematic biopsy prostate cores. Designs D and E allowed for sufficient data extraction of the systematic biopsy alone, MRI plus targeted and systematic biopsy, and MRI plus targeted biopsy pathways, except for the study by Panebianco et al,^27^ which did not separately report the content of targeted and systematic biopsy prostate cores. Randomized clinical trials with design E performed targeted biopsies on the basis of digital rectal examination or ultrasonography findings, which may have resulted in an improved prostate cancer detection in the systematic biopsy alone pathway compared with other study designs.

Several prebiopsy MRI pathways were used in the studies included in this analysis (Figure 3). In all RCTs, individuals with a clinical suspicion of PCa were randomly allocated to either the systematic biopsy alone group or to a prebiopsy MRI group. In all but 1 RCT,^26^ individuals with negative prebiopsy MRI findings proceeded to undergo a systematic biopsy, with this procedure being identical to that performed in the systematic biopsy alone group because there was no visible MRI lesion to be sampled by a targeted approach. In 2 RCTs,^26,29^ individuals with positive MRI findings underwent a targeted procedure alone (MRI plus targeted biopsy pathway), whereas in the other RCTs,^25,27,28,30,31^ individuals with positive MRI findings underwent a combined procedure incorporating both targeted and systematic cores (Figure 3). For 3 of the MRI plus targeted and systematic biopsy RCTs,^25,28,30^ it was possible to extract sufficient data regarding the content of the targeted cores. In 2 RCTs,^25,28^ targeted cores were also taken in patients within the systematic biopsy alone group if suspicious lesions were visible at ultrasonography or palpable during digital rectal examination (Figure 3), which may have increased PCa detection in the control group of these RCTs compared with the systematic biopsy alone group of other RCTs.

Two RCTs^25,29^ used a 1.5-T MRI scanner, 4 RCTs^27,28,30,31^ used a 3.0-T MRI scanner, and 1 RCT^25^ included data from both 1.5- and 3.0-T MRI scanners. Three RCTs^26,27,29^ used a phased-array coil with or without an endorectal coil, 1 RCT^30^ used body and spine matrix surface coils, 1 RCT^31^ used a transrectal coil, 1 RCT^25^ did not use a coil, and 1 RCT^28^ did not report whether a coil was used. One RCT^25^ used biparametric MRI, whereas the other RCTs used mpMRI. Different definitions were used to define a positive MRI, including a Prostate Imaging Reporting and Data System score of 3 or higher,^25,26,29^ Prostate Imaging Reporting and Data System score of 4 or higher,^31^ or any lesion detected at MRI without the use of a standardized reporting system (eTable 2 in the Supplement).^27,28,30^ The images were interpreted by at least 1 experienced radiologist^25,26,31^ or were assessed in consensus by 2 radiologists^27,28,30^ or 3 radiologists^29^ (eTable 1 in the Supplement).

Individuals in the prebiopsy MRI group with positive MRI findings underwent a targeted biopsy. The number of cores sampled during this targeted procedure varied considerably among RCTs (eTable 1 in the Supplement). For example, in 2 RCTs,^25,27^ a maximum of 2 cores were taken per targeted biopsy, whereas in another RCT,^26^ a maximum of 4 cores were obtained from a maximum of 3 areas, resulting in 1 to 12 cores per targeted procedure. The individuals randomized to the systematic biopsy alone group underwent a standard TRUS-guided prostate biopsy systematically sampling the peripheral zones of the prostate gland (eTable 1 in the Supplement) with 12 cores,^25,29^ 14 cores,^27^ or 10 to 12 cores^26,28,30^ taken during the procedure. Most trials used only the transrectal approach to perform targeted prostate biopsies;^25,27,28,30,31^ however, 2 trials^26,29^ used either the transrectal or transperineal approach depending on local expertise or the anatomic location of the radiological lesion. Transperineal approaches were used only in the MRI group of these studies, whereas in the systematic biopsy alone group, all biopsies were performed using the transrectal approach. Furthermore, the manner in which the prebiopsy MRI findings were used to guide the targeted biopsy varied among RCTs. Four RCTs^25,26,29,31^ used MRI-ultrasonography image fusion, 3 RCTs^26,28,30^ used cognitive guidance, and 1 RCT^27^ did not report the method of biopsy guidance.

Individuals received a diagnosis of csPCa, clinically insignificant PCa, or no PCa, depending on the biopsy pathologic results. The characterization of biopsy-detected PCa as being clinically significant or insignificant depended on the Gleason sum score (≥6 or 7), maximum cancer core length (≥3 or 5 mm), and/or the number of positive cores. (With the Gleason scoring system, pathologists grade the cell patterns in the biopsy sample from 1 to 5, where grade 1 cells resemble normal prostate tissue and grade 5 are high-risk cancerous cells. The Gleason score is calculated by adding the grade of the most predominant pattern with the second-most predominant pattern, such as 3 + 4.) However, no 2 studies used the same definition of csPCa (eTable 1 in the Supplement).

Five RCTs^25,26,29,30^ were judged to have a low overall risk of bias (eTable 3 in the Supplement). Two RCTs^27,31^ were judged to have some concerns regarding the randomization process, one of which^27^ did not report methods of randomization, allocation concealment, or baseline characteristics of each group; the other RCT^31^ did not report sufficient information to asses randomization.

**Figure 4.**
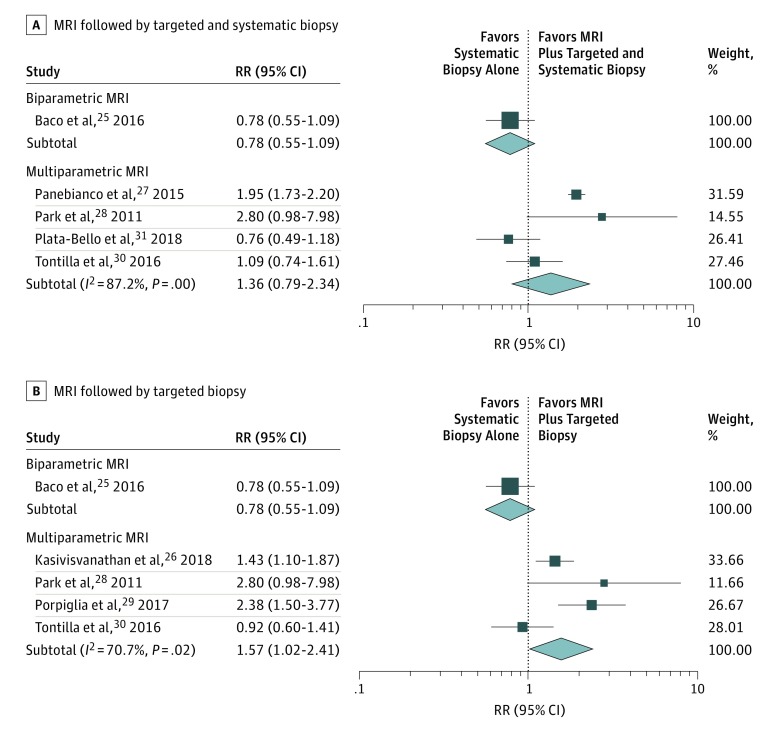
Detection Rate of Clinically Significant Prostate Cancer Risk ratios (RRs) are represented by boxes, with the size of each box representing its weight. Horizontal lines represent 95% CIs. Diamonds represent combined-effect estimates and their 95% CIs. MRI indicates magnetic resonance imaging.

Data from 5 RCTs^24,27,28,30,31^ contributed to the analysis of the MRI plus targeted and systematic biopsy pathway, and data from 5 RCTs^25,26,28,29,30^ were used to analyze the MRI plus targeted biopsy pathway (Figure 4). In 1 study,^25^ the use of prebiopsy biparametric MRI did not significantly improve the detection of csPCa compared with the use of systematic biopsy alone (risk ratio, 0.78; 95% CI, 0.55-1.09). However, in 4 of the RCTs,^26,28,29,30^ the MRI plus targeted biopsy pathway improved the detection of csPCa by 57% (95% CI, 2%-141%; risk ratio, 1.57; [95% CI, 1.02-2.41]; *I*^2^ = 71%) compared with systematic biopsy alone. Compared with systematic biopsy alone, the MRI plus targeted and systematic biopsy pathway did not significantly improve the detection of csPCa (risk ratio, 1.36; 95% CI, 0.79-2.34; *I*^2^ = 87%) in 4 RCTs.^27,28,30,31^

Direct comparison between the 2 prebiopsy MRI pathways, using the 3 RCTs^25,28,30^ that evaluated the MRI plus targeted and systematic biopsy pathway and reported separate data for the targeted and systematic cores regarding PCa detection, showed mixed results. In 2 of these RCTs,^25,28^ the additional acquisition of systematic cores did not identify additional csPCa cases beyond those detected in the targeted cores alone. However, in the study by Tonttila et al,^30^ 4 csPCa cases would have been missed if only a targeted approach had been used (ie, the MRI plus targeted biopsy pathway), which would have resulted in underdiagnosis in 10% of patients with positive MRI findings.

In most RCTs, it was not possible to assess the risk of complications associated with the targeted biopsy procedure compared with systematic TRUS-guided biopsy, because the individuals in the prebiopsy MRI group underwent systematic sampling during the targeted biopsy procedure. In only 2 RCTs^26,29^ was the acquisition of targeted cores not combined with systematic sampling. However, the RCT by Porpiglia et al^29^ is ongoing, and there are plans to report on complications in future publications. Kasivisvanathan et al^26^ reported fewer overall complications for individuals in the prebiopsy MRI group compared with individuals in the systematic biopsy alone group. The frequency of hematuria (30% vs 63%), hemoejaculate (32% vs 60%), rectal bleeding (14% vs 22%), erectile dysfunction (11% vs 16%), and pain at the site of the procedure (13% vs 23%) were each reported to be lower in individuals in the prebiopsy MRI pathway compared with the systematic biopsy alone group.^26^ However, in the prebiopsy MRI pathway, this RCT used both transperineal and transrectal approaches and only transrectal biopsies in the systematic biopsy alone group, which may account for the reduced complications in the MRI pathway. Moreover, approximately one-half of individuals in the prebiopsy MRI group did not undergo a biopsy at all (in the context of the MRI findings being negative); therefore, this would naturally have reduced the risk of complications in this group of the study.

We calculated the percentage of individuals for whom a biopsy was avoided, or could theoretically have been avoided, if the men with mpMRI-negative findings had not undergone prostate biopsy. The percentage of men who may have avoided a biopsy procedure ranged from 23%^27^ to 55%,^31^ with an overall estimate of 33% for all 7 RCTs^25,26,27,28,29,30,31^ (95% CI, 23%-45%; *I*^2^ = 91.8; eFigure 1 in the Supplement). In 6 RCTs,^25,26,27,28,29,30^ the MRI plus targeted biopsy pathway would also theoretically have reduced the number of biopsy cores taken per procedure by 77% (95% CI, 60%-93%) compared with the systematic biopsy alone group. The median number of targeted cores ranged from 1 to 6, compared with a mean number of systematic biopsy cores in the systematic biopsy alone group ranging from 11 to 12.

Overall, 31% (95% CI, 15%-49%; *I*^2^ = 87%) of PCa cases were not visualized at prebiopsy mpMRI in 5 RCTs^27,28,29,30,31^ (eFigure 2A in the Supplement), and most were classified as clinically insignificant (according to a systematic biopsy^28,29,30,31^ or saturation biopsy^27^). In these 5 RCTs,^27,28,29,30,31^ the risk of a patient having csPCa and a negative MRI findings ranged between 0% and 23% (eFigure 2B in the Supplement).

## Discussion

This systematic review and meta-analysis demonstrates that the use of prebiopsy mpMRI combined with a targeted biopsy is superior to a systematic biopsy alone in diagnostic pathways for PCa. This improvement is seen in terms of increased detection of csPCa and a reduced number of biopsy cores obtained during a biopsy procedure, potentially preventing unnecessary biopsies and possibly reducing the overall burden of adverse effects from the invasive biopsy procedure. This observation adds to the evidence suggesting that the incorporation of prebiopsy MRI should be recommended for diagnostic pathways for suspected PCa. Obtaining systematic cores in addition to the targeted cores during a biopsy procedure did not seem to improve detection of csPCa, and only a few PCas were missed. However, data in this area were sparse, and studies may have been underpowered to test this, whereas some level of misclassification could not be ruled out.

To our knowledge, this is the first systematic review to compare 2 MRI pathways (MRI plus targeted and systematic biopsy and MRI plus targeted biopsy) with a pathway including systematic biopsy alone. The main strength of this review is that the inclusion criteria were limited to RCTs, which permits direct comparison between 2 diagnostic pathways with clinically relevant outcomes, as opposed to diagnostic cohort studies that can only inform us about test accuracy measures. Furthermore, all included trials were of high quality with low risk of bias, and there were sufficient data to conduct a meta-analysis on each MRI pathway. Extracting data for both MRI pathways from within the MRI plus targeted and systematic biopsy group of some RCTs allowed for direct comparisons between these pathways, even though none of the RCTs was designed to compare these 2 pathways per se.

### Limitations

Limitations of this meta-analysis include the fact that we were unable to assess publication bias or perform a meta-regression analysis to test for variables associated with PCa detection because of insufficient data. The design of the included studies did not allow for calculation of test properties, such as sensitivity and specificity, because most patients did not undergo a reference standard procedure (ie, saturation biopsy or prostatectomy). Test accuracy measures were beyond the scope of this review, but a systemic review will be published soon.^32^

Two RCTs^27,29^ did not use identical biopsy approaches for all patients in both study groups. Some patients in the prebiopsy MRI group underwent biopsy using a transperineal approach, whereas all patients in the systematic biopsy alone group underwent biopsy using a transrectal approach. The transrectal approach can be less adequate than the transperineal approach in terms of sampling the apex and anterior regions of the prostate. Some of the MRI-guided biopsies were performed through the transperineal approach, which permits better sampling of the apex and anterior regions of the prostate gland. This may have inflated the cancer detection rates in the prebiopsy MRI group. However, because of the limited number of RCTs included, it was not possible to perform a sensitivity analysis on the type of biopsy approach used.

An important limitation of the included RCTs was that each study used a different definition of csPCa, and it was not possible to extract sufficient data for a standardized definition. This may explain the high degree of heterogeneity among studies, which means that results should be interpreted with some caution. Another source of variation was the guidance method used during the biopsy procedure itself. Cognitive guidance is potentially more error prone than MRI-ultrasonography image fusion guidance,^33^ and the 2 RCTs^28,30^ using cognitive guidance missed the highest percentage of csPCa. Only 1 RCT^26^ reported data on complications associated with biopsy; therefore, we have very limited data for this important outcome. None of the RCTs reported long-term follow-up data to capture screening-relevant outcomes, such as time to mortality or cancer-associated mortality.

There have been concerns about the financial costs of MRI, but these have reduced over time, and 2 recent studies^34,35^ based on US and UK data have demonstrated that incorporating MRI can be cost-effective, especially because doing so may avoid some unnecessary biopsies and reduce the burden of overtreatment. Another concern has been the availability of the necessary expertise to interpret MRI scans and perform MRI-guided biopsies. Training is necessary for radiographers to perform high-quality mpMRI scans and for radiologists and urologists to interpret the images and perform targeted biopsies. Standardized reporting has reduced variation in the interpretation of MRI scans among radiologists, but this variation is still significant.^36^ Inaccurate sampling has been identified as a contributor to reduced MRI performance, even in those individuals undergoing MRI-ultrasonography fusion prostate biopsy.^37^

## Conclusions

A key issue in the diagnosis and treatment of PCa remains the need to identify clinically significant disease that requires intervention and to avoid the unnecessary diagnosis of low-risk, low-volume disease. This systematic review and meta-analysis suggests that introducing prebiopsy mpMRI followed by a targeted biopsy into a PCa detection pathway may lead to the performance of fewer biopsies than a pathway using systematic biopsy alone. Such an approach may increase the likelihood of detecting csPCa, while reducing the detection of low-risk tumors. Introducing prebiopsy MRI, therefore, has the potential to transform practice. One RCT^26^ has demonstrated that this may lead to fewer complications, and further studies have indicated that this may be a useful cost-effective strategy. There remain concerns that some csPCa cases may be missed in individuals with an increased age-specific PSA level and negative MRI findings. Combining the MRI results with other measures, such as PSA density (ie, PSA levels adjusted for prostate volume), can potentially decrease the risk of missing these csPCa cases,^38^ but there are few studies in this area, and this requires further investigation. Moreover, there is no evidence regarding the impact of a delayed diagnosis of csPCa after a decision not to perform a biopsy is made on the basis of normal MRI findings in the context of an increased PSA level. The availability of mpMRI and radiologists and urologists trained to use it appear to be the only hurdles to overcome in establishing mpMRI and targeted biopsy with standardized reporting as the recommended diagnostic pathway for men with suspected PCa.

## Notes

Supplement. **eFigure 1.** The Percentage of Patients in the Prebiopsy MRI Arm With a Negative MRI Result **eFigure 2. **Prostate Cancers Missed by Prebiopsy MRI Pathways **eTable 1. **Definitions Used for Clinically Significant Prostate Cancers and Biopsy Methods in Reported Studies **eTable 2. **Multiparametric Magnetic Resonance Imaging **eTable 3. **Risk of Bias Assessment
