# Supplementary material for: Comparison of Multiparametric Magnetic Resonance Imaging and Targeted Biopsy With Systematic Biopsy Alone for the Diagnosis of Prostate Cancer: A Systematic Review and Meta-analysis
Source: JAMA Netw Open. 2019 Aug 7;2(8):e198427. doi: 10.1001/jamanetworkopen.2019.8427 (PMC6686781; doi:10.1001/jamanetworkopen.2019.8427)
Supplement: Supplement. — eFigure 1. The Percentage of Patients in the Prebiopsy MRI Arm With a Negative MRI Result eFigure 2. Prostate Cancers Missed by Prebiopsy MRI Pathways eTable 1. Definitions Used for Clinically Significant Prostate Cancers and Biopsy Methods in Reported Studies eTable 2. Multiparametric Magnetic Resonance Imaging eTable 3. Risk of Bias Assessment [file jamanetwopen-2-e198427-s001.pdf]

Supplementary Online Content

Elwenspoek MMC, Sheppard AL, McInnes MDF, et al. Comparison of multiparametric magnetic resonance imaging and targeted biopsy with systematic biopsy alone for the diagnosis of prostate cancer: a systematic review and meta-analysis. *JAMA Netw Open*. 2019;2(8):e198427. doi:10.1001/jamanetworkopen.2019.8427

eFigure 1. The Percentage of Patients in the Prebiopsy MRI Arm With a Negative MRI Result

eFigure 2. Prostate Cancers Missed by Prebiopsy MRI Pathways

eTable 1. Definitions Used for Clinically Significant Prostate Cancers and Biopsy Methods in Reported Studies

eTable 2. Multiparametric Magnetic Resonance Imaging

eTable 3. Risk of Bias Assessment

This supplementary material has been provided by the authors to give readers additional information about their work.

eFigure 1. The Percentage of Patients in the Prebiopsy MRI Arm With a Negative MRI Result

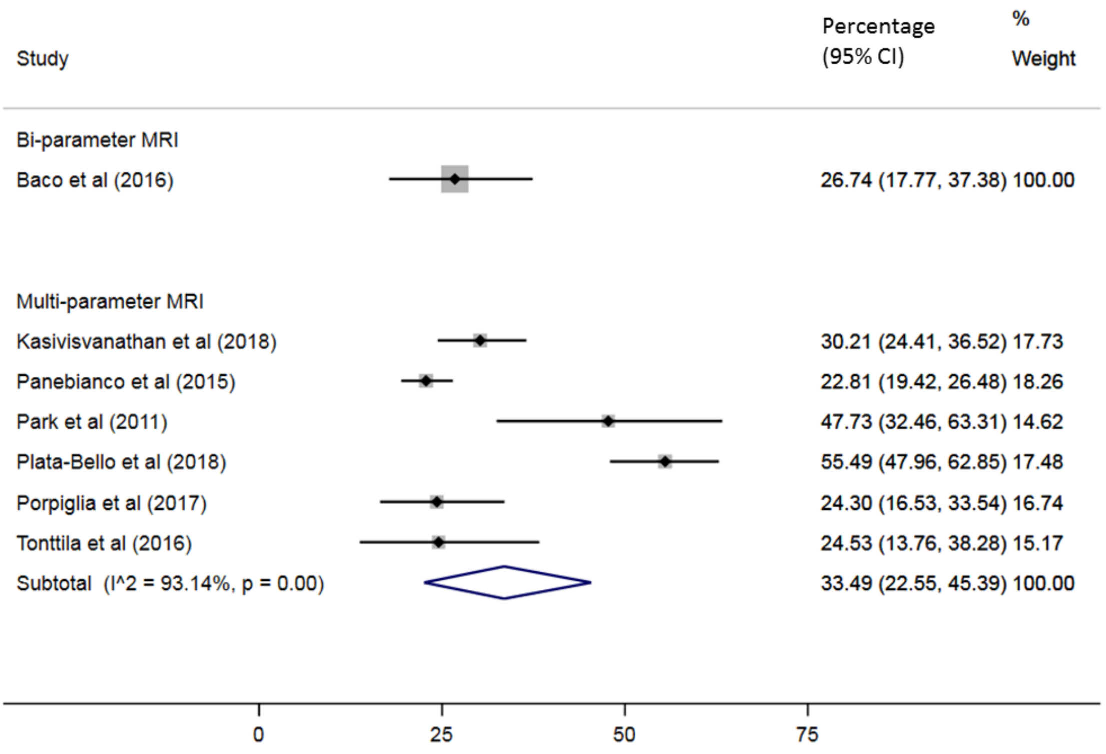

If the pre-biopsy MRI would be used as a triage test, these patients would avoid a biopsy procedure, although some prostate cancers will be missed (see eFigure 2).

© 2019 Elwenspoek MMC et al. *JAMA Network Open.*

eFigure 2. Prostate Cancers Missed by Prebiopsy MRI Pathways

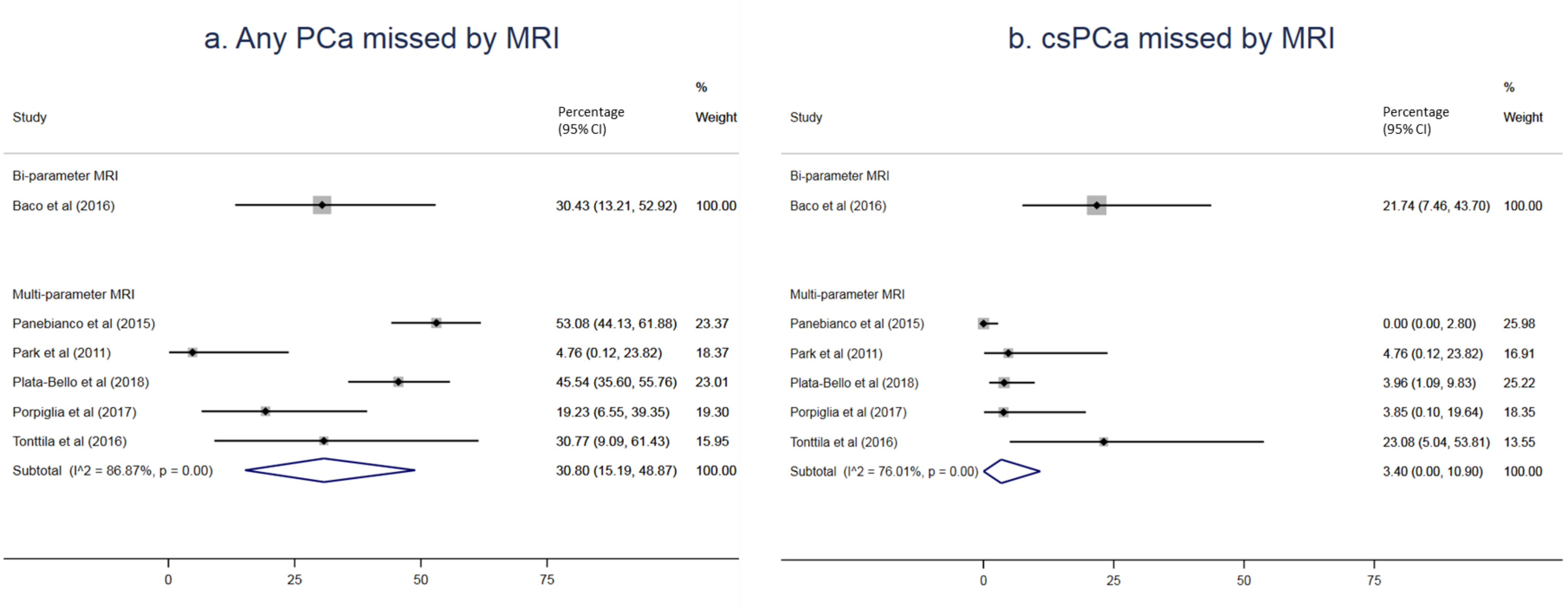

Percentage of patients with a “negative MRI” where at subsequent TRUS-guided systematic biopsy any-grade prostate cancer (a) or clinically significant prostate cancer (b) was detected.

© 2019 Elwenspoek MMC et al. *JAMA Network Open.*

eTable 1. Definitions Used for Clinically Significant Prostate Cancers and Biopsy Methods in Reported Studies

Study	Definition clinically significant	Biopsy in standard pathway	Targeted biopsy in MRI pathway
Baco (2016),[20]<br>Norway	Max CCL ≥5 mm for Gleason score 6 disease or any max CCL for Gleason score ≥7 disease	12 cores<br>Transrectal approach<br>Ultrasound-guided<br>Systematic biopsy	2 cores<br>Transrectal approach<br>MRI-US image fusion guidance<br>Targeted biopsy
Kasivisvanathan (2018),[21]<br>UK	Presence of a single biopsy core indicating disease of GS ≥7	10-12 cores<br>Transrectal approach<br>Ultrasound-guided<br>Systematic biopsy	Maximum of 4 cores were obtained from a maximum of 3 areas suggestive of PCa<br>Transrectal or transperineal approach<br>Cognitive or MRI-US image fusion guidance (according to local expertise)<br>Targeted biopsy
Panebianco (2015),[22]<br>Italy	Not reported*	14 cores<br>Transrectal approach<br>Ultrasound-guided<br>Systematic biopsy	2 cores from the two areas most suggestive of PCa<br>Transrectal approach<br>Not reported under what guidance<br>Targeted biopsy
Park (2011),[23]<br>Korea	Gleason score ≥6 and "histologically confirmed with adenocarcinoma"	10-12 cores<br>Transrectal approach<br>Ultrasound-guided<br>Systematic biopsy	Maximum of 3 cores<br>Transrectal approach<br>Cognitive guidance<br>Targeted biopsy
Plata-Bello (2018),[26]<br>Spain	Any Gleason score ≥7 or CCL ≥5 mm	Number of cores not reported<br>Transrectal approach<br>Ultrasound-guided<br>Systematic biopsy	Number of cores not reported<br>Transrectal approach<br>MRI-US image fusion guidance<br>Targeted biopsy
Porpiglia (2017),[24]<br>Italy	Any Gleason score ≥7 or max CCL ≥5 mm	12 cores<br>Transrectal approach<br>Ultrasound-guided<br>Systematic biopsy	3-6 cores were obtained from a maximum of 2 areas suggestive of PCa<br>Transrectal or transperineal approach (based on the location of the lesion)<br>MRI-US image fusion guidance<br>Targeted biopsy
Tonttila (2016),[25]<br>Finland	Gleason score ≥7, more than two positive cores, or max CCL ≥3 mm	10-12 cores<br>Transrectal approach<br>Ultrasound-guided<br>Systematic biopsy	1-2 cores were obtained from a maximum of 2 areas suggestive of PCa<br>Transrectal approach<br>Cognitive guidance<br>Targeted biopsy

*Definition used to extract data: number of csPCa = number of patients who received treatment; number of non-significant PCa = number of patients who received active surveillance
Abbreviations: CCL, cancer core length; csPCa, clinically significant prostate cancer; GS, Gleason score; PCa, prostate cancer; SB, standard biopsy; TRUS, transrectal ultrasound; US, ultrasound.

© 2019 Elwenspoek MMC et al. *JAMA Network Open.*

eTable 2. Multiparametric Magnetic Resonance Imaging

Study	Field strength of the magnet	Coils used (e.g. pelvic, endorectal)	Sequences used (parameters)	Reporting method used	Definition of positive MRI results	Experience radiologist interpreting MRI images
Baco (2016),[20] Norway	1.5-T	None used	bpMRI:<br>T2-weighted and DWI	PI-RADS v1	PI-RADS score ≥3	The images were interpreted by one radiologist with experience of greater than 1000 MRI-prostate at the start of this study
Kasivisvanathan (2018),[21] UK	1.5-T or 3.0-T	Pelvic phased-array coil, with or without endorectal coil	mpMRI:<br>T2-weighted, DCE, and DWI	PI-RADS v2	PI-RADS score ≥3	Median of 5 years of experience (IQR 4.5-10)<br>Median of 300 prostate MRIs reported per year (IQR 200-500)
Panebianco (2015),[22] Italy	3.0-T	Phased-assay coil and endorectal coil	mpMRI:<br>T2-weighted, DCE, and DWI	PI-RADS v1	"Suspicious lesion detected by mpMRI"	The images were evaluated in consensus by 2 genitourinary radiologists, with 13 and 4 years of experience
Park (2011),[23] Korea	3.0-T	Not reported	mpMRI:<br>T2-weighted, DCE, and DWI	Not reported	A target visualized by any of the MRI parameters	The images were assessed by consensus by two radiologists with more than 7 years of experience
Plata-Bello (2018),[26] Spain	3.0-T	Transrectal coil	mpMRI:<br>T1- and T2-weighted, DCE, and DWI	PI-RADS (unclear which version)	PI-RADS score ≥4	Images were interpreted by a radiologist with >10 years of experience in prostate MRI
Porpiglia (2017),[24] Italy	1.5-T	32-channel phase array coil or four-channel phase array coil combined with an endorectal coil	mpMRI:<br>T2-weighted, DCE, and DWI	PI-RADS v1	PI-RADS score ≥3	Three experienced radiologists analyzed the mpMRI findings (level of experience not reported)
Tonttila (2016),[25] Finland	3.0-T	Body and spine matrix surface coils	mpMRI:<br>T1-weighted axial and T2-weighted triplanar, DCE, and DWI	Scores 1-4, indicating likelihood of cancer	Any target visible on MRI (score 2-4)	The images were independently interpreted by two experienced body radiologists

Abbreviations: bpMRI, biparametric MRI; DCE, dynamic contrast-enhanced; DWI, diffusion-weighted imaging; IQR, interquartile range; mpMRI, multi-parametric MRI; PI-RADS, prostate imaging reporting and data system.

© 2019 Elwenspoek MMC et al. *JAMA Network Open.*

eTable 3. Risk of Bias Assessment

Study	Randomization process	Deviations from intended interventions	Missing outcome data	Measurement of the outcome	Selection of the reported result	Overall Bias
Baco (2016),[20] Norway	Low	Low	Low	Low	Low	Low
Kasivisvanathan (2018),[21] UK	Low	Low	Low	Low	Low	Low
Panebianco (2015),[22] Italy	Some concerns	Low	Low	Low	Low	Some concerns
Park (2011),[23] Korea	Some concerns	Low	Low	Low	Low	Low
Plata-Bello (2018),[26] Spain	Some concerns	Some concerns	Some concerns	Low	Low	Some concerns
Porpiglia (2017),[24] Italy	Low	Low	Low	Low	Low	Low
Tonttila (2016),[25] Finland	Low	Low	Low	Low	Low	Low

© 2019 Elwenspoek MMC et al. *JAMA Network Open.*

© 2019 Elwenspoek MMC et al. *JAMA Network Open.*
